# Development of clinical-guideline-based mobile application and its effect on head CT scan utilization in neurology and neurosurgery departments

**DOI:** 10.1186/s12911-022-01844-3

**Published:** 2022-04-20

**Authors:** Zahra Meidani, Fatemeh Atoof, Zohre Mobarak, Ehsan Nabovati, Reza Daneshvar Kakhki, Ebrahim Kouchaki, Esmaeil Fakharian, Ali Mohammad Nickfarjam, Felix Holl

**Affiliations:** 1 Health Information Management Research Center, Kashan University of Medical Sciences, Kashan, Iran; 2 Department of Biostatistics & Epidemiology, Faculty of Health, Kashan University of Medical Sciences, Kashan, Iran; 3 Department of Health Information Management and Technology, Faculty of Allied Medical Sciences, Kashan University of Medical Sciences, Kashan, Iran; 4 Autoimmune Diseases Research Center, Kashan University of Medical Sciences, Kashan, Iran; 5 Department of Neurology, Faculty of Medicine, Kashan University of Medical Sciences, Kashan, Iran; 6 Trauma Research Center, Kashan University of Medical Sciences, Kashan, Iran; 7 DigiHealth Institute, Neu-Ulm University of Applied Sciences, Neu-Ulm, Germany; 8 Institute for Medical Information Processing, Biometry, and Epidemiology, University of Munich, Munich, Germany

**Keywords:** Mobile applications, Cell phone, Intention, Physicians, Medical record, Tomography X-ray computed

## Abstract

**Background:**

There is little evidence regarding the adoption and intention of using mobile apps by health care professionals (HCP) and the effectiveness of using mobile apps among physicians is still unclear. To address this challenge, the current study seeks two objectives: developing and implementing a head CT scan appropriateness criteria mobile app (HAC app), and investigating the effect of HAC app on CT scan order.

**Methods:**

A one arm intervention quasi experimental study with before/after analysis was conducted in neurology & neurosurgery (N&N) departments at the academic hospital. We recruited all residents' encounters to N&N departments with head CT scan to examine the effect of HAC app on residents' CT scan utilization. The main outcome measure was CT scan order per patient for seven months at three points, before the intervention, during the intervention, after cessation of the intervention -post-intervention follow-up. Data for CT scan utilization were collected by reviewing medical records and then analyzed using descriptive statistics, Kruskal-Wallis, and Mann-Whitney tests. A focus group discussion with residents was performed to review and digest residents' experiences during interaction with the HAC app.

**Results:**

Sixteen residents participated in this study; a total of 415 N&N encounters with CT scan order, pre-intervention 127 (30.6%), intervention phase 187 (45.1%), and 101 (24.3%) in the post-intervention follow-up phase were included in this study. Although total CT scan utilization was statistically significant during three-time points of the study (*P* = 0.027), no significant differences were found for CT utilization after cessation of the intervention (*P* = 1).

**Conclusion:**

The effect of mobile devices on residents' CT scan ordering behavior remains open to debate since the changes were not long-lasting. Further studies based on real interactive experiences with mobile devices is advisable before it can be recommended for widespread use by HCP.

## Background

Mobile devices and mobile health applications (mHealth apps) are among the fastest and convenient ways for physicians to access educational materials, including e-books, drug information, and clinical guidelines [1–3]. One of the areas in which guideline-based mobile apps can assist physicians is the CT scan appropriateness guidelines, which have been developed following the steady CT utilization increase. The number of CT examinations increased globally more than doubled between 1988 and 2008 [4]. The massive volume of imaging not only imposes a tremendous cost to the health care system, but unnecessary exposure to radiation also contributes in increased risk of development of cancer [5]. Regardless of these adverse outcomes, it is argued that a substantial of imaging procedures may be unnecessary [6]. Imaging guideline known as appropriateness criteria presented as one of the strategies to change physician CT scan utilization behavior. Previous evidence suggested that implementing imaging appropriateness guideline and education decrease the rate of performance of inappropriate imaging examinations [7, 8]. Making guidelines available at the point of care via information technology (IT) including guideline-based handhelds was proposed as an effective strategy to improve performance of healthcare providers and reduce unnecessary medical procedures [9]. Qumseya [10] presented that (87%) believed that access to relevant guidelines at the point of care would improve guideline adherence. The majority of physicians agreed that medical apps facilitate their access to clinical practice guidelines [11]. Under these capabilities, there is growing interest in the potential of medical apps among health care professionals. Accordingly, we hypothesized that appropriateness guideline-based mobile apps affect head CT utilization. However, earlier studies debated that employing IT approach clinical guidelines were ineffective or had a modest impact on CT utilization [12, 13], and contributing factors in the successful adoption of mobile apps in clinical practice are still in jeopardy [14]. There is no clear understanding of the motivations and interests which affect physicians to adopt and continuous use mobile apps [15]. Previously reported results indicated that mobile apps' adoption by HCP narrowly focused on evaluating attitudes and perceptions and has limitations in terms of evaluating impacts [16–18]. Although some medical apps are already available for the use of HCP, there is a lack of knowledge regarding the successful adoption of mobile apps among physicians. Székely [19] introduced nearly 102 mobile apps for radiology by the year 2012 that some of them like ACR appropriateness criteria app and eRoentgen Radiology DX have been developed by major stakeholders in mobile apps stores such as Apple, Google, Microsoft and blackberry to assist physicians in selecting appropriate imaging procedure based on patients' conditions [20, 21]. However, it has been reported that healthcare providers are not involved in the development and evaluation of these mobile apps [22]; and the evaluation of apps is limited to some app reviews provided at the mobile app stores [21].

Thus, understanding the factors that influence healthcare professionals' adoption of the mobile apps bring potential benefits for patients, health care decision-makers, and main stakeholders in mobile app stores. To consider this challenge, we seek two objectives: (1) developing and implementing a Head CT scan Appropriateness Criteria mobile app (HAC app) for residents CT scan ordering; (2) investigating the effect of HAC app on CT scan utilization via before and after the intervention.

## Methods

### Study setting

The current study was launched in neurology & neurosurgery (N&N) departments of the academic hospital with 510 beds affiliated to Kashan University of Medical Sciences (KAUMS), Iran. Given university has been focused on a series of studies to integrate cost information of health care services into the physicians' education (cost consciousness) and decline inappropriate medical investigations [23–25], including medical imaging, laboratory tests, bed utilization, etc. among health care providers [26–29].

### Study design

We performed one arm intervention quasi experimental study with before/after analysis to examine the effect of HAC app on CT scan utilization for all encounters to N&N departments. Following the performed study on the CT utilization at a given hospital [26] in 2017, we aimed at evaluating an intervention to implement the clinical-guideline-based mobile application on head CT utilization at N&N departments from May 2018 to November 2018. Phases of the present study included: (1) development and implementing of HAC app; (2) investigating the effect of HAC app on head CT utilization via before- after intervention, and post intervention follow up phase; (3) and assimilation of residents' real experience during the interaction with the HAC app via focus group discussion FGD.

### Development of HAC App

#### HAC app building approach

To select a system-building approach for developing HAC app, a multidisciplinary expert panel consisting of physicians, and professionals from health information sciences were formed. Since the complexity and the size of information technology projects would increase the likelihood of its failure [30], the research team adopted an affordable, iterative process via prototyping approach. To consider the highlighted role of providing access to relevant guidelines at the point of care on guideline adherence by physicians [10, 11], functional requirements for HAC app mainly focused on supporting electronic access to Care Core guideline. As far as we know, the evidence to investigate the physicians' engagement with mobile application in daily clinical practice is meager and there was a lack of knowledge regarding to: (1) conducting this type study in terms of study protocol and (2) anticipating the outcome of mobile app intervention in terms of its effectiveness.

We started this project as a pilot project with basic requirements and the minimum changes on the existing clinical processes. Design phase of HAC app involves an understanding of the residents' basic needs and requirements for ordering a Head CT scan through two focus group discussions (FGD). Selecting appropriateness guidelines for ordering Head CT scans also was discussed in this phase. All free clinical guidelines for ordering Head CT scans were collected [31–36] and discussed via interactive FGD with physician trainees (resident) and attending physicians. Table 1 indicates the list of appropriateness guideline for imaging. The Care Core National Guideline was confirmed as a CT scan guideline for adoption in HAC app since physicians believed it was close to their medical textbooks.

**Table 1:** Imaging appropriateness criteria/guideline

Title of the appropriateness criteria/guideline	Developed by
American College of Radiology (ACR) CT Scan Appropriateness Criteria [31]	American College of Radiology (ACR)
Diagnostic Imaging Referral Guidelines: a guide for physicians [32]	The Canadian Association of Radiologist
Clinical Appropriateness Guidelines [33]	American Imaging Management (AIM)
Care Core Criteria for Imaging [34]	Care Core National
Referral Guidelines for Imaging [35]	European Commission
Referral Guidelines [36]	Roya College of Radiologist

The CT scan appropriateness guidelines extracted through a literature search was presented to the expert panel [31–36]. They were invited to discuss on most suitable CT scan appropriateness guidelines and HAC app requirements for developing an electronic guideline based on mobile application. Decision was made based on interaction between the researchers and experts, and considering suitability of the guideline for clinical processes and the clinical training materials of the residents.

A trained moderator (researcher) explained the different phases of the study and the non-evaluative environment of the FGD. The FGD moderator has the following responsibilities: encouraging all residents to participate actively, summarizing and extracting the main ideas from the comments. We analyzed collected data and transformed physicians' ideas into meaning units relevant to the study questions.

#### HAC App content and functionality

We applied 4-tier architecture including presentation layer, data service layer, business logic layer, and data access layer to develop the HAC app. The HAC app's graphical user interface was designed on the Android 4.1 or higher using the JavaScript language. HAC app owns offline capability which allows the user to run the HAC app regardless of internet connectivity. It does not contain, maintain, receive, or send patient information. The HAC app encompasses entire basic criteria arranged by Care Core guideline for head CT scan. Care Core provides a list for disease titles for example head trauma, which are supplemented by the list of clinical criteria in terms of signs and symptoms of the given disease. Under each main heading or in front of each condition the appropriate imaging procedure in the form of MRI, CT, computed tomography angiography (CTA) is provided. The main objectives of the first phase were to inform the residents that HAC app is the preliminary pilot system solely focusing on the main processes of ordering CT examinations. It was also emphasized that HAC app might need to be refined based on their inputs and investigating its effectiveness on CT utilization.

### Investigating the effectiveness of HAC app on head CT utilization: before and after intervention

#### Intervention

The before and after intervention phases of the study were conducted for five months from May 2018 to September 2018 for two months baseline audit/ pre-intervention and three months the intervention phase. Since the timing of the before and after measurements is important in quasi- experimental study, researcher leave one month for forming and stabilizing the new behaviors; therefore, the intervention phase is extended to three months.

Although performing a case–control study increased its robustness and generalizability of our findings, a before- after intervention was conducted due to the following limitations:

- The size of the hospital: Shahid Beheshti is a medium size hospital with small number of residents (n = 16). So, the number of residents were not enough to conduct case and control study.
- Limited number of existing academic hospitals: Shahid Beheshti hospital is the only academic hospital in the given city and it was impossible to consider other neurology and neurosurgery departments as a control group. Selecting the attending physicians as a control group were also impossible due to imperfect matching criteria in terms of age, working experiences, expertise, and knowledge among residents and attending physicians. Selecting another clinical department at Shahid Beheshti hospital was not also logical since a control group might suffer from contamination bias. We believed that members of the ‘control’ group might inadvertently be exposed to the intervention.

To protect the physician against the patient as a potential plaintiff and fear of litigation, using the HAC app was optional. The residents were unaware about the objective of the study and outcome measurement (effect on HAC app on number of CT scan ordered). No other competing interventions were conducted to reduce head CT scan for hospitalized patients at N&N departments. However, applying the HAC app as an assistive device to order head CT was emphasized by attending physicians. To audit and track residents adopting the HAC app, the research team audited CT scan utilization and communicated the rate of CT ordered by residents with the chair of the N&N departments during the intervention phase.

The engagement of senior physicians was crucial for HAC app adoption. Using the HAC app was an option thus, we need a strategy to motive residents to use HAC app. Moreover, the attending's emphasis was a part of the adoption process of HAC app. Evidence introduced social support (senior/peer pressure) as one of the main features of the early stages of mobile engagement [37].

### Investigating the continuous use of HAC app

To realize the continuous use of HAC app, we ceased the intervention; therefore, no further tracking of the residents of CT scan utilization was performed nor was it communicated with the chair of the N&N departments. Then, residents continued the use of HAC app and CT utilization were investigated two months after cessation of the intervention from October 2018 to November 2018.

#### Selection of participants

All residents in N&N departments (n = 16) who used Android mobile phones were included in the current study since literature debated that the residents were mostly responsible for ordering inappropriate services at the academic hospitals [23–25]. All encounters, were hospitalized in the N&N departments and received CT scan during study periods were used for outcome measure.

#### Outcome measure

According to previous studies to investigate the effectiveness of information technology tools on physicians' ordering behavior [38], per patient CT scan ordered by residents was applied as a unit of analysis during research phases. The outcome measure was assessed using a checklist as well as reviewing medical records at all two-time points of the intervention. In the baseline phase, all medical records, which belonged to patients with head CT scan and were hospitalized at N&N departments, were reviewed using a two-section checklist. The first section covered patient background information, including age, sex, and patient record number, length of stay, medical diagnosis, type of treatment, and insurance type. The second section covered background information about head CT, such as type of CT (CTA, with contrast, without contrast), requested ward (neurology, neurosurgery), time and date of CT scan, and the numbers of CT scans. The same chart review process was applied during three months of intervention phase and two months after cessation of the intervention or post-intervention follow up phase.

### Understanding residents' experience with the HAC app

We conducted one FGD with residents to provide insights and further guidance for developers on contributing factors in the successful adoption of HAC app. The research team aimed two objectives to develop FGD: (1) to review and digest residents' experience acquired during interaction with the HAC app; (2) to pave the way to go beyond narrow considerations of the IT artifact and to get deepen into underexplored facts for further revision of HAC app via iterative approach. FGD was directed with semi-structured questions to prompt discussion on the HAC app and the usage of mobile apps in general. These sessions were digitally recorded and transcribed by two authors to identify common themes. Quotations illustrating the key themes were analyzed based on physicians' insights.

### Data analysis

Data were analyzed using descriptive statistics such as frequency and median. Since the distribution of data was skewed or non-normally distributed data, we applied the median to report CT utilization per patient during three points of the study. A Chi-square test was used to compare the difference between the CT scans of two groups of N&N, and the exact test was used if the test conditions were not met. Chi-squared test is used to determine the significant relationship between sex, disease, and treatment of encounters during three phases of the study. Since the distribution of age and length of stay were not normal, the Kruskal–Wallis test was used to determine any significant relationships between age and length of stay throughout various stages in this study. To determine statistically significant differences for CT scan utilization between three phases of study also we also used Kruskal-Wallis test. The Mann-Whitney test is used to compare CT scan utilization significant differences between two phases of study independently.

All statistical analyses were performed using Statistical Package for Social Sciences 16.0 (SPSS Inc., Chicago, IL, USA) at a significant level of 0.05. We applied Wolcott [39] thematic analysis approach including description, analysis, and interpretation to analyze FGD collected data and transform physicians' ideas into insights, and themes.

### Ethical consideration

The current study was approved by a Research Ethics Committee of Deputy of Research & Technology at KAUMS [Code# IR.KAUMS.MEDNT.REC.1396.95]. We declare that all research phases performed in accordance with the Declaration of Helsinki – Ethical Principles for Medical Research Involving Human Subjects. Research participants were informed about the study before initiating the intervention phase. Informed consent was obtained and the voluntary nature of the participation was explained to participants, and they were assured about anonymity and confidentiality of data collected.

## Results

### Development of HAC App: content and functionalities

**Fig. 1:**
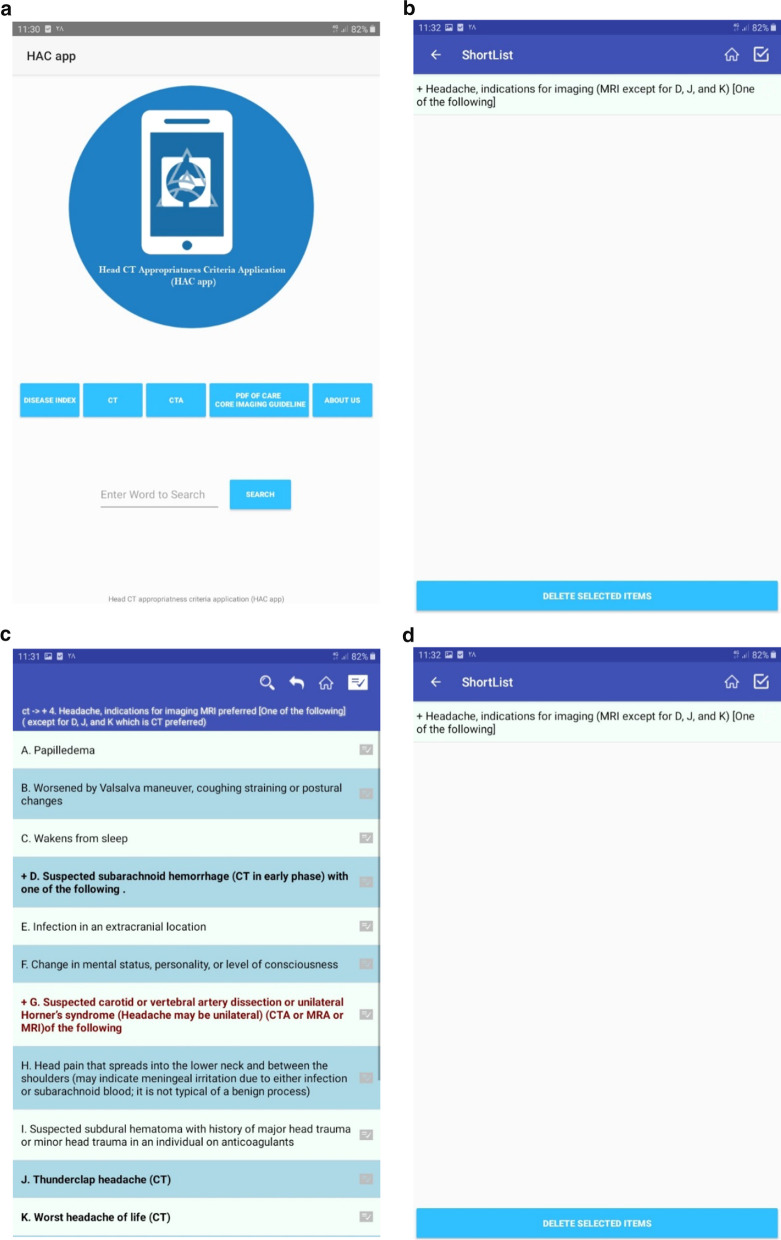
Screenshots of HAC app

HAC app enables end-users to search head CT scan appropriateness criteria based on diseases, signs and symptoms, and modality type including CT scan, CTA, and MRI. To apply HAC app, users enter specific diseases, signs or symptoms enclosed in Care Core guideline in the "Index" box. To avoid confusion and proper data presentation, the list of clinical criteria under the disease heading are grouped using plus sign (+). The detailed clinical criteria are provided via clicking on the plus sign. HAC app also supports searching conditions under headings of the imaging modalities. To organize and easily find frequently used diseases or clinical criteria, a shortlist menu is designed. It enables users to add common diagnoses to the shortlist menu. Screenshots of the functionalities of the HAC app are presented in Fig. 1.

### Investigating encounters with CT scan at N&N departments

A total of 415 N&N encounters with CT scan order, pre-intervention 127 (30.6%), intervention phase 187 (45.1%), and 101 (24.3%) in the post-intervention follow-up phase were included in this study. Of these encounters, 298 (71.8) were male, and 117 (28.2) were female. The patients' median age was 54 (Q_1_ = 31, Q_3_ = 73). The patients were mostly admitted at the N&N wards due to hemorrhagic lesion 94 (22.65%), ischemic lesions 92 (22.17%), and other neurologic disorders 127 (30.60%).

**Fig. 2:**
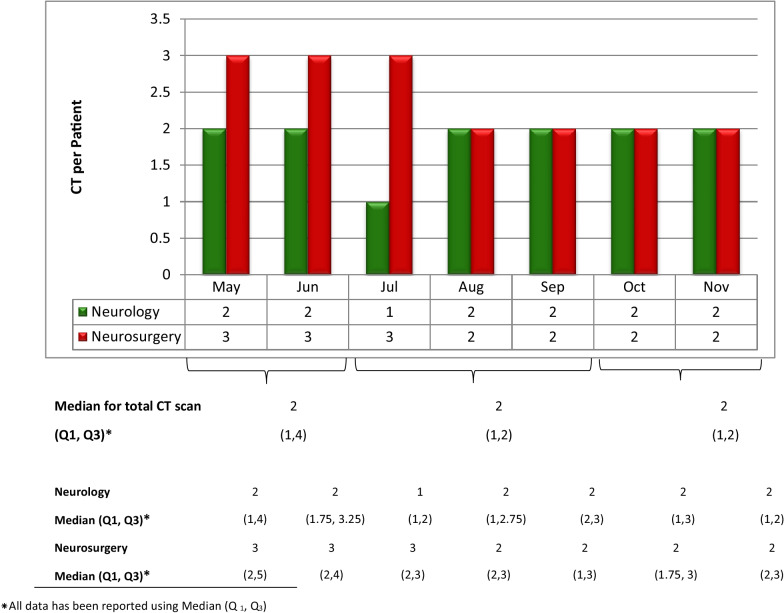
Comparisons of monthly CT scan utilization per patient at N&N departments

Table 2 also indicates that the maximum number of patients who needed CT scan 187 (45.1%) were admitted during the intervention phase. Moreover, complicated cases including patients with the final diagnosis of hemorrhagic lesion 47 (50%) and ischemic lesions 54 (58.7%) also increased during the intervention phase. However, the comparison of CT scan ordering per patient at the N&N department did not increase during the intervention phase from July to September (Fig. 2).

**Table 2:** Patient characteristics during three phases of pre-intervention, intervention, and post-intervention

Patient characteristics	Pre-intervention (N = 127)	Intervention (N = 187)	Post-Intervention (N = 101)	Total (N = 415)
Sex (%)				
Male	88 (29.5)	138 (46.3)	72 (24.2)	298 (100)
Female	39 (33.3)	49 (41.9)	29 (24.8)	117 (100)
Diagnosis (%)				
Head trauma	25 (36.2)	29(42)	15 (21.8)	69 (100)
Hemorrhagic lesion	27 (28.7)	47 (50)	20 (21.3)	94 (100)
Ischemic lesions	18 (19.6)	54 (58.7)	20 (21.7)	92 (100)
Tumors	15 (45.5)	8 (24.2)	10 (30.3)	33 (100)
Other neurological disorders	42 (33.1)	49 (38.6)	36 (28.3)	127 (100)
Treatment (%)				
Surgical	61 (39.6)	55 (35.7)	38 (24.7)	154 (100)
Medical	66 (25.3)	132 (50.6)	63 (24.1)	261 (100)
Age				
Median (Q1; Q3)*	47 (26; 67)	58 (37; 76)	55 (33; 70)	54 (31; 73)
Length of stay				
Median (Q1; Q3)*	8 (5; 12)	6 (4; 10)	5 (4; 10)	6 (4; 10)

*All data has been reported using Median (Q_1_, Q_3_)

### Investigating the effectiveness of HAC app on head CT utilization: before and after intervention

We compared monthly CT scan utilization per patients during three phases of study using median. Although Fig. 2 reveals the median for CT scan utilization during three phases of study was 2, third quartiles (Q3) indicates CT scan utilization declines from median= 4 at the before intervention phase to 2 after the intervention phase. This decline (Q3=2) remained the same during the intervention phase and post-intervention follow up phase.

According to Table 3, total head CT utilization for seven months was 920. We investigated the effect of HAC app on CT utilization at all three-time points of the intervention including pre-intervention, intervention phase, and post- intervention follow up phase using Kruskal–Wallis test. The decrease of total CT scan utilization at three phases of study at both N&N departments was statistically significant (*P* = 0.027).

**Table 3:** CT scan utilization per patient in three phases of pre-intervention, intervention, and post- intervention follow up phase

Phases of study	CT scan utilization						
	Total head CT		CT at Neur.		CT at Neurosurg.		Total
	Total	Per Pt	Total	Per Pt	Total	Per Pt	
Pre-intervention	334	2 (1,4)*	64	0 (0,1) *	272	2 (1,4) *	336
Intervention	381	2 (1,2)*	150	0 (0,1) *	231	1 (0,2) *	381
post-intervention follow up	205	1 (1,2)*	73	0 (0,1) *	130	1 (0,2) *	203
Total	**920**		**287**		**633**		**920**
*p*-value Kruskal-Wallis	0.027		0.028		˂ 0.001		
*p*-Value Mann–Whitney							
Pre-intervention and intervention	0.054		0.024		˂0.001		
Pre and post intervention follow up	0.075		0.35		0.003		
Intervention and post intervention follow up	1		1		1		

*Pt* patient, *Neur* neurology, *Neurosurg* neurosurgery

*All data has been reported using Median (Q_1_, Q_3_)

To compare CT scan utilization significant differences between two phases of study independently, Mann-Whitney test was used. Significant differences were found in the pre-intervention phase and intervention phase at both neurology (*P* = 0.024) and neurosurgery (*P* ˂ 0.001) departments. However, the decline was marginally significant for total CT scan utilization (*P* = 0.054).

### Understanding the continuous use of HAC app

To understand continuous intention of use of HAC app among residents, we compared the CT scan utilization during the intervention phase and post- intervention follow up phase (Table 3). The total head CT scan utilization and CT scan utilization at neurology and neurosurgery departments were not statistically significant between two phases of intervention phase and post- intervention follow up phases (*P* = 1).

### Understanding residents' experience with the HAC app

We reviewed and digested residents' experience acquired during interaction with the HAC app via FGD and two critical themes extracted in terms of HAC app strengths and area for improvement, and physicians' expectations of HAC app next version (Table 4).

**Table 4:** Thematic analysis of physicians' insights regarding HAC app

Physicians' insights	Themes	Quotations
Current version of HAC app: strengths & weaknesses	User interface	"I think the icons are really clear."
		"HAC app was easy to use"
		"The visibility of screen was appropriate."
		"Help tab was unclear; because it was located at the bottom of “About us” tab."
	Usefulness	"Providing an electronic guideline with a capability to search would support its accessibility; however, it is not efficient to be used at patient bedside".
		The navigation between diagnosis, symptoms, and pages was a bit awkward."
		"It was suitable for reading not practical at the point of care; Actually, at the point of care, I need something which speeds up my workflow and productivity."
		"We mostly need mobile devices for prompt decision making. So, it is apparent that in these kind of situations we do not have time to search and read something. We seek suggestions, instead".
	Functionality	"It is easy to know what to do/where to move to perform tasks."
		"Lack of proper search capabilities: once the search was made, the term was highlighted in the app (e.g. headache); providing a long list of conditions which enclose the term "Headache" make it confusing."
		"Lack of information layering; since the mobile screen is too small, providing a long list of search results make it time demanding and inefficient."
		"Unclear status: “not entirely clear if I actually completed the task”, since there is no feedback if you complete the task."
		"I believe the small size of the mobile screen makes it difficult to work with; In fact, searching information from a long list of symptoms or diseases in the form of a mobile LCD was hard and time-consuming."
Physicians' expectations of HAC app next version	Effectiveness	"Maybe developing a disease-specific app which focuses on CT scan appropriateness criteria for one or two diseases be more helpful."
		"I prefer app to assist me in the prediction of the next step for patient treatment; something could help me for decision-making, for example, interpreting the CT scan."
		"Perhaps rather than providing an electronic guideline with a capability to search from the list of disease or symptoms, providing a kind of algorithm for complex neurological conditions would provide more clinical value."
		"I prefer those applications that I can enter the patient's signs and symptoms and it lets me know which imaging procedure is suitable for the given patient."

## Discussion

The purpose of the present study was to examine the effect of clinical-guideline-based mobile application, HAC app on head CT scan utilization in N&N departments. Our result demonstrated that guideline-based mobile application might affect the head CT scans utilization during the intervention period. The results of this study are inconsistent with those of Carnevale and Sharp's study of using clinical guidelines through information technology (IT) tools. In their research, clinical guidelines in the form of a computerized decision support system (CDSS) had no or modest effect on reducing CT scans [12, 13]. However, the results of present study are consistent with those of Bookman, Ip, Goergen, and Min [40–43]. The reason for this discrepancy may be related to how an intervention is implemented and reinforced. In all of the mentioned studies, including the present study, there is one thing in common: the varieties of organizational, administrative, and social approaches in the form of feedback, social pressure like emphasis by senior physicians, and hard stop approaches which were employed to integrate IT tools in clinical practices and workflows. For example, Goergen designed the CDSS fully interactive to answer physicians' questions and to provide feedback, decision reasoning, and interpretation [42]. Therefore, it appears that adopting mobile devices, like any other IT application, should integrate all the socio-technical requirements in terms of human, social, organizational, and technical factors to adopt successfully [44]. The study also revealed that although HAC app affects residents' CT scan utilization, these changes were not sustained. As a result, we cannot directly attribute the resident's CT utilization changes to HAC app, and there might be other contributing factors to these results. We believe that the HAC app was effective during the intervention phase, since using guideline and reducing inappropriate CT utilization was also highlighted by senior physicians in the morning report sessions. Therefore, this emphasis might work as a subjective norm/social influence for adopting mobile app. Previous evidence argued that subjective norms/social influence, including "superior influence", "peer influence", and "regulation" has a substantial effect on intention of use of IT application [45, 46]. Existing literature also highlighted the crucial role of social support/ senior pressure in the early stages of mobile engagement model [37].

Another purpose of the current study was to understand the continuous intention of use of HAC app among residents. The results suggested that the decrease of the CT utilization was statistically insignificant in post- intervention follow up phase and residents discontinued using HAC app. In our opinion, one of the reasons that the residents stopped using the HAC app after removing the intervention might have been attributed to its functionality and perceived usefulness. Previous evidence indicates that effectiveness, efficiency, functionality, usability, and quality of mobile application had significant effect on its acceptance [47–50]. There has been a focus in the literature on the strong impact of perceived usefulness on intention to use mobile application. Payne found physicians employ mobile apps by which they improve care efficiency and productivity [51]. Pokhrel [52] via a qualitative study among HCP presented that they prefer mobile apps which support them in their clinical practices including "suggestive diagnosis and treatment after entering the symptoms of a particular disorder", and "guides and supports in diagnosis and treatment". Our finding during FGD with residents also support Pokhrel's finding and emphasize that the sole automation of clinical guideline using mobile app would not entirely address HCP's needs at the point of the care. Our results do not support the results derived by Qumseya [10], Al-Ghamdi [11], Dupaix [17], and Hakes [53]. In his study, Al-Ghamdi debated that physicians introduced a great impact of mobile devices on clinical practices through faster access to clinical practice guideline [11]. Dupaix et al, via survey study, also revealed that distribution of mobile devices among the residents would increase reading educational material among physicians and their study time at the hospital [17]. This difference may be due to the fact that aforementioned studies mainly focused on evaluating attitudes and perceptions of HCP regarding mobile devices not evaluating their actual use at the point of care. It would be a huge gap among HCP's perceptions about the capabilities of mobile devices and real interaction with them through clinical workflow.

### Implications for research and education

Given the short term effect of HAC app on CT utilization our results did not necessarily confirm any direct cause-and-effect relationship between the mobile app and physicians' behavior. Lack of the results from the empirical studies in the area of effectiveness of mobile apps among HCP and focus of existing literature on evaluating attitude and perception of HCP necessitate the further studies to realize contributing factors in the success of mobile adoption and its continuous usage. It seems mobile apps' investigations should move towards its second wave of studies in the form of experimental, clinical trial, cohort studies at both phases of adoption and mobile engagement. Moreover, in order to increase robustness of the future research design and reduce results discrepancies, developing studies protocol to support more reliable and consistent results for these type of studies is strongly recommend. Since, mobile artefact considers as one of the most widespread technology adoptions of all time, the Lesson learned from these sorts of studies creates values for variety of stakeholders.

### Strengths and limitations

To our knowledge, this study represents the first attempt to investigate the appropriateness guideline-based mobile application to support head CT scan ordering. While most previous studies concerned physicians' attitudes and perceptions towards mobile application, we have conducted an experimental one to evaluate the impacts of mobile apps on care outcome. Our study has several limitations: First, it was carried out on a small sample size for a relatively short time period in a single health care setting. Such limitations decrease the generalizability of findings to other similar groups. However, we worked post-intervention follow-up to make it entirely impossible to confirm any cause-and-effect relationship of the mobile app on CT order by chance. In the area of the development of mobile application, HAC app's graphical user interface was designed only for the Android mobile operating system. Moreover, using the HAC app was optional and statistics reports of HAC app for instance how long users spent in the app, how many times applied the app, and which icons, buttons are clicked have not be tracked in the current study. To eliminate the effect of usability factors in HAC app adoption by residents, we should have conducted usability testing before full implementation of HAC pp.

Although, the attending's emphasis was a part of the adoption process of HAC app, their influence should have been investigated on the use of the HAC app. Moreover, audits of care processes at the N&N departments for example residents' consultation with attending physicians for ordering CT scan was also not carried out in the current study. The outcome assessment of HAC app was narrowly focused on number of CT scan, and various features of CT scan ordering including repeated CT scan, CT scan intervals and their appropriateness were not accurately measured in the study. Furthermore, Hence patients' medical diagnosis would affect CT scan requests attention should be given to patients' recruitment in the forthcoming studies.

## Conclusion

Hence, the effect of guideline -based mobile application was not a long-lasting change, it did not necessarily confirm any cause and effect relationship between the mobile app and physicians' behavior. We suggested further studies using a larger sample and in the form of experimental, clinical trial, and post-implementation studies before its widespread use**.**

## Data Availability

Authors declares that all data and material will be available for further research and verification by contacting corresponding author.

## References

[1] Chase TJG, Julius A, Chandan JS, Powell E, Hall CS, Phillips BL, Burnett R, Gill D, Fernando B (2018). Mobile learning in medicine: an evaluation of attitudes and behaviours of medical students. BMC Med Educ..

[2] Bonabi M, Mohebbi SZ, Martinez-Mier EA, Thyvalikakath TP, Khami MR (2019). Effectiveness of smart phone application use as continuing medical education method in pediatric oral health care: a randomized trial. BMC Med Educ..

[3] Ventola CL (2014). Mobile devices and apps for health care professionals: uses and benefits. P T..

[4] Ngoya PS, Muhogora WE, Pitcher RD (2016). Defining the diagnostic divide: an analysis of registered radiological equipment resources in a low-income African country. Pan Afr Med J..

[5] Zhou JC, Zheng SW, Yu YX, Rouleau K, Jiang WL, Jin CW, Zhou DY, Pan KH, Yu YS (2012). Trends in computed tomography utilization and association with hospital outcomes in a Chinese emergency department. PLoS ONE..

[6] Zare S, Mobarak Z, Meidani Z, Nabovati E, Nazemi Z (2022). Effectiveness of clinical decision support systems on the appropriate use of imaging for central nervous system injuries: a systematic review. Appl Clin Inform..

[7] Levy G, Blachar A, Goldstein L, Paz I, Olsha S, Atar E, Goldberg A, Bar Dayan Y (2006). Nonradiologist utilization of American College of Radiology Appropriateness Criteria in a preauthorization center for MRI requests: applicability and effects. AJR Am J Roentgenol..

[8] Tahvonen P, Oikarinen H, Pääkkö E, Karttunen A, Blanco Sequeiros R, Tervonen O (2013). Justification of CT examinations in young adults and children can be improved by education, guideline implementation and increased MRI capacity. Br J Radiol..

[9] Taba P, Rosenthal M, Habicht J (2012). Barriers and facilitators to the implementation of clinical practice guidelines: a cross-sectional survey among physicians in Estonia. BMC Health Serv Res.

[10] Qumseya B, Goddard A, Qumseya A, Estores D, Draganov PV, Forsmark C (2021). Barriers to clinical practice guideline implementation among physicians: a physician survey. Int J Gen Med..

[11] Al-Ghamdi S (2018). Popularity and impact of using smart devices in medicine: experiences in Saudi Arabia. BMC Public Health..

[12] Carnevale TJ, Meng D, Wang JJ, Littlewood M (2015). Impact of an emergency medicine decision support and risk education system on computed tomography and magnetic resonance imaging use. J Emerg Med..

[13] Sharp AL, Huang BZ, Tang T, Shen E, Melnick ER, Venkatesh AK, Kanter MH, Gould MK (2018). Implementation of the Canadian CT head rule and its association with use of computed tomography among patients with head injury. Ann Emerg Med..

[14] Sondaal SF, Browne JL, Amoakoh-Coleman M, Borgstein A, Miltenburg AS, Verwijs M, Klipstein-Grobusch K (2016). Assessing the effect of MHealth interventions in improving maternal and neonatal care in low- and middle-income countries: a systematic review. PLoS ONE.

[15] Hsiao JL, Chen RF (2019). Understanding Determinants of health care professionals' perspectives on mobile health continuance and performance. JMIR Med Inform..

[16] Koohestani HR, Soltani Arabshahi SK, Fata L, Ahmadi F (2018). The educational effects of mobile learning on students of medical sciences: a systematic review in experimental studies. J Adv Med Educ Prof..

[17] Dupaix J, Chen JJ, Chun MB, Belcher GF, Cheng Y, Atkinson R (2016). The effect of mobile tablet computer (iPad) implementation on graduate medical education at a multi-specialty residency institution. Hawaii J Med Public Health..

[18] Liu CF, Cheng TJ (2015). Exploring critical factors influencing physicians' acceptance of mobile electronic medical records based on the dual-factor model: a validation in Taiwan. BMC Med Inform Decis Mak..

[19] Székely A, Talanow R, Bágyi P (2013). Smartphones, tablets and mobile applications for radiology. Eur J Radiol..

[20] https://www.imedicalapps.com/2020/07/acr-appropriateness-criteria-app-review/.

[21] https://www.imedicalapps.com/2009/10/eroentgen-app-reviewed-but-is-it-worth/.

[22] Aungst TD, Clauson KA, Misra S, Lewis TL, Husain I (2014). How to identify, assess and utilise mobile medical applications in clinical practice. Int J Clin Pract..

[23] Vegting IL, van Beneden M, Kramer MH, Thijs A, Kostense PJ, Nanayakkara PW (2012). How to save costs by reducing unnecessary testing: lean thinking in clinical practice. Eur J Intern Med..

[24] Tartaglia KM, Kman N, Ledford C (2015). Medical student perceptions of cost-conscious care in an internal medicine clerkship: a thematic analysis. J Gen Intern Med..

[25] Iwashyna TJ, Fuld A, Asch DA, Bellini LM (2011). The impact of residents, interns, and attending's on inpatient laboratory ordering patterns: a report from one university's hospitalist service. Acad Med..

[26] Meidani Z, Hamidian Y, Farzandipour M, Aliasgharzade A (2017). CT utilization: a case study in Iran based on ACR appropriateness criteria. Radiol Manage..

[27] Meidani Z, Farzandipour M, Farrokhian A, Haghighat M (2016). A review on laboratory tests' utilization: a trigger for cutting costs and quality improvement in health care settings. Med J Islam Repub Iran..

[28] Meidani Z, Mousavi GA, Kheirkhah D, Benar N, Maleki MR, Sharifi M, Farrokhian A (2017). Going beyond audit and feedback: towards behavior-based interventions to change physician laboratory test ordering behaviour. J R Coll Physicians Edinb..

[29] Meidani Z, Farzandipour M, Hosseinpour M, Kheirkhah D, Shekarchi M, Rafiei S (2017). Evaluating inappropriate patient stay and its reasons based on the appropriateness evaluation protocol. Nurs Midwifery Stud.

[30] Pan G, Hackney R, Pan SL (2008). Information systems implementation failure: insights from prism. Int J Inf Manag..

[31] American College of Radiology (ACR) CT scan appropriateness criteria. 2012 [cited 2018]. https://www.acr.org/Clinical-Resources/ACR-Appropriateness-Criteria.

[32] The Canadian Association of Radiologist: Diagnostic Imaging Referral Guidelines: a guide for physicians. 2005 [cited 2017]. https://car.ca/patient-care/referral-guidelines/.

[33] AIM Clinical Appropriateness Guidelines for Radiology. 2013 [cited 2018]. https://aimspecialtyhealth.com/resources/clinical-guidelines/radiology/.

[34] Care Core Criteria for Imaging. 2015 [cited 2017]. www.qualchoice.com › media › qualchoice-carecore-ima.

[35] Referral guidelines for imaging European Commission. 2000 [cited 2017]. ec.europa.eu › energy › sites › ener › files › documents

[36] Referral guidelines by the Royal College of Radiologists (RCR). 2010 [cited 2017]. https://www.rcr.ac.uk/clinical-radiology/being-consultant/rcr-referral-guidelines/about-irefer.

[37] Burns K, Nicholas R, Beatson A, Chamorro-Koc M, Blackler A, Gottlieb U (2020). Identifying mobile health engagement stages: interviews and observations for developing brief message content. J Med Internet Res..

[38] Meidani Z, Nabovati E, Zare S, Moosavi GA, Masoud A, Omidvar A, Holl F (2021). Effectiveness of an automated feedback with dashboard on use of laboratory tests by neurology resident. Inform Med Unlocked..

[39] Wolcott HF (2002). Writing up qualitative research…better. Qual Health Res..

[40] Bookman K, West D, Ginde A, Wiler J, McIntyre R, Hammes A, Carlson N, Steinbruner D, Solley M, Zane R (2017). Embedded clinical decision support in electronic health record decreases use of high-cost imaging in the Emergency Department: EmbED study. Acad Emerg Med..

[41] Ip IK, Raja AS, Gupta A, Andruchow J, Sodickson A, Khorasani R (2015). Impact of clinical decision support on head computed tomography use in patients with mild traumatic brain injury in the ED. Am J Emerg Med..

[42] Goergen SK, Fong C, Dalziel K, Fennessy G (2006). Can an evidence-based guideline reduce unnecessary imaging of road trauma patients with cervical spine injury in the emergency department?. Australas Radiol..

[43] Min A, Chan VWY, Aristizabal R, Peramaki ER, Agulnik DB, Strydom N, Ramsey D, Forster BB (2017). Clinical decision support decreases volume of imaging for low back pain in an urban emergency department. J Am Coll Radiol..

[44] Baxter Gordon, Sommerville Ian (2011). Socio-technical systems: from design methods to systems engineering. Interact Comput..

[45] Fishbein M, Ajzen I (1976). Belief, attitude, intention, and behavior: an introduction to theory and research.

[46] Chang TK, Huang H, Chang SM. Understanding educational administrators' subjective norms on their use intention toward on-line learning. In: Uden L, Herrera F, Bajo Pérez J, Corchado Rodríguez J, editors. 7th international conference on knowledge management in organizations: service and cloud computing. advances in intelligent systems and computing, vol 172. Berlin: Springer; 2013. 10.1007/978-3-642-30867-3_24.

[47] Ehrler F, Weinhold T, Joe J, Lovis C, Blondon K (2018). A mobile app (BEDSide Mobility) to support nurses' tasks at the patient's bedside: usability study. JMIR Mhealth Uhealth..

[48] Geerds MAJ, Nijmeijer WS, Hegeman JH, Vollenbroek-Hutten MMR (2020). Mobile app for monitoring 3-month postoperative functional outcome after hip fracture: usability study. JMIR Hum Factors..

[49] Akour H. Determinants of mobile learning acceptance: an empirical investigation in higher education. In: Conference proceedings. 2010.

[50] Almaiah MA, Alismaiel OA (2019). Examination of factors influencing the use of mobile learning system: an empirical study. Educ Inf Technol.

[51] Payne KB, Wharrad H, Watts K (2012). Smartphone and medical related App use among medical students and junior doctors in the United Kingdom (UK): a regional survey. BMC Med Inform Decis Mak..

[52] Pokhrel P, Karmacharya R, Taylor Salisbury T, Carswell K, Kohrt BA, Jordans MJD, Lempp H, Thornicroft G, Luitel NP (2021). Perception of healthcare workers on mobile app-based clinical guideline for the detection and treatment of mental health problems in primary care: a qualitative study in Nepal. BMC Med Inform Decis Mak..

[53] Hakes NA, Kethman WC, Spain D, Nassar AK (2020). Mobile application-based guidelines to enhance patient care and provider education in trauma and acute care surgery. Trauma Surg Acute Care Open..

